# Feasibility and acceptability of transcutaneous tibial nerve stimulation for the treatment of bladder storage symptoms among people with multiple sclerosis

**DOI:** 10.1186/s40814-022-01120-1

**Published:** 2022-07-30

**Authors:** Hawra B. Al Dandan, Rose Galvin, Katie Robinson, Doreen McClurg, Susan Coote

**Affiliations:** 1 School of Allied Health, Faculty of Education and Health Sciences, University of Limerick, Limerick, Ireland; 2 Imam Abdulrahman Bin Faisal University, Dammam, Saudi Arabia; 3 Health Research Institute, University of Limerick, Limerick, Ireland; 4 Aging Research Centre, University of Limerick, Limerick, Ireland; 5 Nursing, Midwifery and Allied Health Professions Research Unit, Glasgow Caledonian University, Glasgow, UK; 6 Physical Activity for Health Research Centre, University of Limerick, Limerick, Ireland; 7 Multiple Sclerosis Society of Ireland, Dublin, Ireland

**Keywords:** Neurogenic lower urinary tract dysfunction, Neuromodulation, Electrical nerve stimulation, Quality of life questionnaire, Tibial nerve

## Abstract

**Background:**

Neurogenic lower urinary tract dysfunction is an abnormality in the presence of underlying neurologic disease. A recent systematic review and meta-analysis demonstrated that storage phase symptoms are the predominant symptoms among people with multiple sclerosis (PwMS). Transcutaneous tibial nerve stimulation (TTNS) is a non-invasive treatment for bladder storage symptoms; however, the potential efficacy of stimulation among PwMS is based on a small number of studies with the absence of high-quality evidence. The aim of this study was to evaluate the feasibility, acceptability, and safety of TTNS in PwMS using an affordable transcutaneous electrical nerve stimulation (TENS) unit.

**Methods:**

A total of 23 participants with MS enrolled in the study. The primary outcomes included recruitment/retention rate, completion of the outcomes and the intervention, adherence to the protocol, adverse events, and acceptability of the intervention. The primary outcomes were assessed using diaries and a satisfaction questionnaire. The secondary outcomes included changes in urinary symptoms and quality of life assessed using a set of validated outcome measures including a 3-day bladder diary, PPIUS, ICIQ-OAB, and KHQ at baseline and post-intervention.

**Results:**

Twenty participants completed the study. Three participants (13.04%) withdrew. All 20 participants completed the 6-week intervention and all the outcome measures (100%), with no reported adverse events. Participants were satisfied and found the unit acceptable. Three-day bladder diary showed changes in urinary frequency from a daily median of 10 times to 8 times and daily median urgency changed from 6 times at baseline to 2 times post-intervention. PPIUS showed changes in daily median sever urgency from 3 points (IQR=4) to 1 point (IQR=1) post-intervention. ICIQ-OAB total scores changed from 8 points (IQR=2.25) to 4 points (IQR=2.5) post-intervention. Median and mean scores of KHQ showed a clinical meaningful change of QoL in part-two and part-three of the questionnaire.

**Conclusions:**

TTNS is feasible, safe, and acceptable for PwMS. Changes of urinary symptoms scores and QoL post-intervention suggested improvements. Future implications need to consider the treatment protocol including frequency of treatment sessions, duration of treatment, and the electrical stimulation parameters as well as the outcome measures followed in the current study for the implementation of the future pilot RCT.

**Trial registration:**

ClinicalTrials.gov NCT04528784. Registered on 27 August 2020. https://register.clinicaltrials.gov/prs/app/action/LoginUser?ts=1&cx=-jg9qo4.

**Supplementary Information:**

The online version contains supplementary material available at 10.1186/s40814-022-01120-1.

## Key messages regarding feasibility

- It was uncertain whether people with multiple sclerosis could be recruited at the required rate for a pilot randomised controlled trial to be conducted in future study. It was uncertain whether Transcutaneous tibial nerve stimulation (TTNS) as an option to manage urinary symptoms could be feasible in terms of acceptability, completion of required outcome measures, and safety of the intervention.
- The intervention of TTNS using an affordable Transcutaneous electrical nerve stimulation (TENS) unit of 3 sessions/week for 6 weeks was found to be feasible, acceptable, and safe with a high adherence rate to the treatment protocol.
- Treatment protocol and the outcome measures followed in the current single-arm feasibility study were recommended for the future pilot RCT.

## Background

Neurogenic lower urinary tract dysfunction (NLUTD) is an abnormality in the presence of underlying neurologic disease [1]. The pattern of lower urinary tract dysfunction following neurological disease depends on the site and nature of the lesion [2, 3]. In people with MS (PwMS), central nervous system lesions are mainly present at supra-pontine and/or supra-sacral areas affecting the storage phase, or both storage and voiding phase accompanied with incomplete bladder emptying [3, 4]. A recent systematic review and meta-analysis demonstrated that storage phase symptoms are the predominant symptoms among PwMS. Urinary frequency had a pooled prevalence of 73.45% followed by urgency at 63.87% using subjective outcome measures [5]. Commonly, patients may present with mixed storage and voiding phase symptoms which was reported in 50–60% of women and men with MS respectively [6].

In PwMS, urinary symptoms negatively impacts on emotional wellbeing, physical health, employment opportunities, recreational activities, and social life [7–11]. Qualitative research has similarly found that urinary symptoms are very troublesome for PwMS and that many PwMS continue to suffer in silence, unsure or unaware of what services to access and they frequently experience gaps in health service provision [12].

Tibial nerve stimulation (TNS) is a form of non-surgical neuromodulation [4] and its main function is to inhibit bladder contraction which in turn reduces high bladder pressure and therefore prevents urinary incontinence [13] and preserve long-term renal function. PwMS and healthcare providers have reported they are open to the use of transcutaneous tibial nerve stimulation (TTNS) for urinary symptoms [12]. A systematic review [14] on the efficacy of TNS among people with MS identified seven studies: 6 focused on percutaneous tibial nerve stimulation (PTNS) and one focused on TTNS. There was significant heterogeneity across the included studies concerning sample sizes, populations studied, the absence of placebo or sham arm and inconsistency relating to the electrical stimulation parameters, frequency of treatment per week, and total number of sessions. The authors concluded that further well-designed experimental studies are needed to examine the impact of TTNS, using a non-invasive technique by applying two adhesive pads rather than the semi-invasive procedure of PTNS by inserting a needle, for neurogenic bladder storage symptoms in PwMS.

The primary aim of the current study is to assess the feasibility and acceptability of TTNS. The secondary aim is to explore changes in bladder storage symptoms and quality of life following treatment

## Methods

### Study design

This is a single-arm feasibility study conducted and reported in line with the CONSORT extension for pilot and feasibility studies [15] (Additional file 1). A detailed protocol for the study is published elsewhere [16].

### Setting and participants

Community-dwelling people with any type of MS in the mid-West of Ireland were eligible to participate in the study if they met the following criteria: ambulatory (with or without assistive device), aged ≥18 years old, presented with at least one bladder storage-related symptom such as urinary frequency, urinary urgency, nocturia, with or without incontinence and were willing to give written informed consent.

### Identification and consent

Recruitment to the study commenced in October 11, 2020, and follow-up was completed in March 12th 2021. Recruitment was conducted using two methods: (1) open recruitment for PwMS through MS Ireland’s communication channels including MS News e-zine, social media and direct email to members of the Mid-West branch of MS Ireland took place between October 2020 and January 2021; (2) a convenience sample of PwMS from existing participants who have already participated in our previous qualitative study [12]. Eligible participants were provided with a link to a survey using Qualtrics Survey Software Platform to read and sign the informed consent form, complete demographic data including the Patient Determined Disease Steps (PDDS) [17] and baseline outcome measures.

Once the baseline data were completed, participants received a downloadable 3- Day bladder diary by email. Participants were provided with a TENS unit and a short demonstration video for the application of TENS, and two downloadable documents: adverse events report diary and adherence to the treatment protocol. A follow-up virtual meeting was held to discuss the application of the unit with all participants. Weekly virtual meetings were also held with all participants to check adherence to the treatment protocol, adverse events, and address any participant queries.

Post-intervention (at the end of week 6), all participants received another link to Qualtrics Survey Software to complete post-intervention measures and a downloadable 3-day bladder diary. Reminders complete measures were sent to all participants within 5 days.

### TTNS Intervention

Description of intervention is presented in Table 1. All TENS units were programmed and locked by KR to ensure fixed parameters were used among participants throughout the intervention. The stimulation frequency was set at 10 Hz [18–20] with a pulse duration of 0.2–0.5 ms equal to 200 μs to allow action potentials to leave the hyperpolarisation zone induced by the anodic phase [19, 21, 22]. A motor response (flexion of the great toe, and fanning of the other toes), alone or with a sensory response, (a tingling sensation at the sole of the foot) indicated the correct placement of electrode which was modified by increasing the current amplitude mA, intensity. Participants applied stimulation in a supine position with extended legs or supported sitting with the knee extended to avoid nerve root compression at the knee joint. The intervention consisted of three sessions per week, 30 min per session for a period of 6 weeks. These parameters (duration of session, frequency of sessions and timing of overall intervention) were based on findings from our qualitative study with PwMS [12], and a previous TTNS trial [23].

**Table 1:** TTNS intervention

**Unit used for stimulation**	Transcutaneous Electrical Nerve Stimulation (TENS)/NeuroTrac Dual channel
**Unit price**	€55.20
**Manufacturer**	Verity Medical Ltd., United Kingdom
**Type of electrodes**	2 self-adhesive electrodes
**Size of electrodes**	VS.5050 50 × 50 mm, square.
**Placement of electrodes**	The positive electrode was placed behind the left medial malleolus, and the negative electrode was located between 5 and 10 cm distally above the medial malleolus

### Outcome measures

The primary outcome for this study was feasibility of the intervention. This was assessed using six different metrics: (1) the number of participants who were recruited to the study, the proportion of those who (2) completed the outcome measures, (3) the 6-week intervention, (4) adhered to the treatment protocol [24], (5) the incidence of adverse events reported during the intervention, and finally, (6) acceptability of the TENS was explored using a self-report questionnaire developed by the researchers containing 5-point Likert scales: 1= “strongly disagree”; 2= “disagree”; 3= “neutral”; 4= “agree”; 5= “strongly agree”.

Secondary outcomes were used to assess the changes in bladder storage symptoms and quality of life using the International Consultation of Incontinence Questionnaire- Overactive Bladder (ICIQ-OAB) [25, 26]. The total score ranges from 0 to 16 with higher values indicating increased urinary symptom severity. Bother scales are not combined in the overall score [27]. A 3-Day bladder diary [28, 29] was used to calculate the episodes of day and night time urinary frequency. The Patient Perception of Intensity of Urgency Scale (PPIUS) [30] was included with 3-day bladder diary. PPIUS was used to assess the severity and intensity of urinary urgency. The total score ranges from 0 to 4 with higher values indicating increased urinary symptoms severity. Quality of life (QoL) was evaluated using the King’s Health Questionnaire (KHQ) [28, 31, 32]. The KHQ has three parts consisting of 21 questions in 10 main domains. Each domain scores range from 0 to 100 points whereas the severity of urinary symptom domain is scored from 0 to 30 points.

### Sample size

As this is a single-arm feasibility study and not designed to evaluate the clinical effectiveness, we did not undertake a formal power analysis for sample size for a primary outcome [33]. We adopted a pragmatic approach to our choice of sample size of 20 participants. It was based on the availability of TENS devices (10 available machines) and the time allocated for the conduct of the study.

### Determining progression to the pilot trial

A priori, our progression criteria to proceed to a pilot trial were determined by exploring if minimum success criteria were achieved in key feasibility aims and objectives including:

- A minimum of 80% recruitment
- A minimum of 80% completion rate of key outcome measures

### Statistical analysis

SPSS software (Version 26.0. Armonk, NY: IBM Corp) was used for statistical analysis. All six measures of feasibility were analysed using descriptive statistics and reported as percentages and proportions. Continuous data were assessed for normality using the Shapiro-Wilks test. Means and standard deviations (SD) are reported where data are normally distributed, and medians and interquartile ranges (IQR) are reported as a conservative estimate where data are not normally distributed. The secondary outcomes of the ICIQ-OAB, KHQ [34] and the 3-day bladder diary data including the episodes of urinary frequency, nocturia, and incontinence as well as the scale of PPIUS were summarised at baseline and post intervention using medians (IQRs) and/or means (SD) depending on the distribution of data. Given the small sample size, comparability tests between baseline and post-intervention were deemed inappropriate [35]

## Results

### Feasibility of intervention

#### Recruitment, retention, and completion rates

**Fig. 1:**
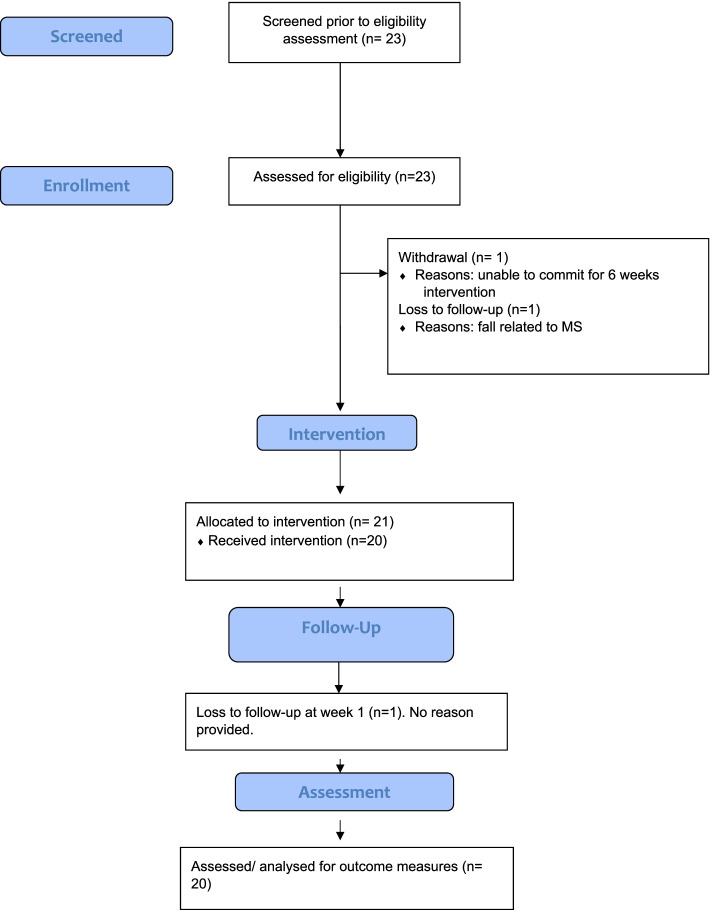
Flow diagram of MS participants

Between October 2020 and January 2021, 25 participants expressed an interest in participating in the study; 23 participants enrolled in the study, 20 participants completed the study Three participants (13.04%) discontinued their participation: one withdrew from the study before commencing the intervention due to inability to commit for 6-week, and two participants were lost to follow-up before commencing the study. All 20 participants who completed the 6-week intervention completed all outcome measures (100%). The flow of study participants is outlined in Fig. 1

Table 2 contains the descriptive characteristics of participants, stratified by those who completed (*n*=20) and those who discontinued/withdrew (*n*=3). Of the 20 participants who completed the study, 6 (30%) were male and 14 (70%) were female. The duration of MS varied from one to 45 years with the onset of LUTSs varied from one to 25 years. The most common type of MS was relapsing remitting (RRMS) (*n*=12, 60%). All participants reported their urinary symptoms as a combination of symptoms (*n*=20, 100%) whereas storage symptoms were reported in seven cases (35%). The most frequently reported symptom was urgency (*n*=16, 80%) followed by frequency (*n*=13, 65%) and nocturia (*n*=12, 60%).

**Table 2:** Participant’s demographic characteristics (*n*=23)

Category	Variable	Participants completed the study (20): ***n*** (%)	Participants lost to follow (3): ***n*** (%)
**Gender**	Female	14 (70%)	2 (66.67%)
	Male	6 (30%)	1 (33.33%)
**Age**	18–24	-	-
	25–34	-	-
	35–44	6 (30%)	2 (66.67%)
	45–54	6 (30%)	1 (33.33%)
	55–64	6 (30%)	-
	65–74	1 (5%)	-
	75–84	-	-
	85 or older	1 (5%)	-
**Marital status**	Single	2 (10%)	2 (66.67%)
	Married	17 (85%)	1 (33.33%)
	Widowed	-	-
	Divorced	1 (5%)	-
	Separated	-	-
**MS type**	RR MS	12 (60%)	2 (66.67%)
	PP MS	5 (25%)	1 (33.33%)
	SP MS	3 (15%)	-
	PR MS	-	-
**MS diagnosis in years**	1–5	2 (10%)	-
	6–10	8 (40%)	1 (33.33%)
	11–15	3 (15%)	2 (66.67%)
	16–20	3 (15%)	-
	21–25	2 (10%)	-
	26–30	1 (5%)	-
	31–35	-	-
	36–40	-	-
	41–45	1 (5%)	-
**LUTSs main**	Urgency	16 (80%)	3 (100%)
	Frequency	13 (65%)	2 (66.67%)
	Nocturia	12 (60%)	1 (33.33%)
	Urge incontinence	6 (30%)	1 (33.33%)
	Terminal dribbles	4 (20%)	-
	Hesitancy	5 (25%)	-
	Feeling of incomplete bladder emptying	8 (40%)	1 (33.33%)
	Urinary incontinence	5 (25%)	2 (66.67%)
	Double void	4 (20%)	1 (33.33%)
**LUTSs in years**	1–5	6 (30%)	1 (33.33%)
	6–10	9 (45%)	-
	11–15	3 (15%)	2 (66.67%)
	16–20	1 (5%)	-
	21–25	1 (5%)	-
**Previous TX**	None	4 (20%)	1 (33.33%)
	Pharmacologic	7 (35%)	1 (33.33%)
	Non-pharmacologic:		
	Exercises	3 (15%)	1 (33.33%)
	Nerve stimulation with another treatment (Exercise and/or Catheterization)	3 (15%)	-
	Behavioural	3 (15%)	-
**Current TX**	None	6 (30%)	2 (66.67%)
	Pharmacologic	9 (45%)	1 (33.33%)
	Pharmacologic with Catheterization	2 (10%)	-
	Non-pharmacologic:		
	Exercises	2 (10%)	-
	Nerve stimulation	-	-
	Behavioural	1 (5%)	-
**Prophylactic**	Yes	3 (15%)	-
	No	17 (85%)	3 (100%)
**Comorbidities**	None	8 (40%)	1 (33.33 %)
	Thyroid related issues	2 (10%)	1 (33.33%)
	Hypertension	1 (5%)	1 (33.33%)
	Reynolds syndrome	1 (5%)	
	Cancer	1 (5%)	-
	Foot drop/mobility	2 (10%)	-
	Migraine	1 (5%)	1 (33.33%)
	hearing impairment	1 (5%)	-
	Bowel related issues/duodenal ulcer	3 (15%)	-
**PDDS-Mobility**	(0) Normal	3 (15%)	-
	(1) Mild disability	1 (5%)	
	(2) Moderate disability	3 (15%)	1 (33.33%)
	(3) Gait disability	2 (10%)	
	(4) Early cane	3 (15%)	
	(5) Late cane	4 (20%)	1 (33.33%)
	(6) Bilateral support	3 (15%)	
	(7) Wheelchair	1 (5%)	1 (33.33%)
	(8) Bedridden	-	

*MS* multiple sclerosis, *RR* relapsing–remitting, *SP* secondary progressive, *PP* primary progressive, *PR* progressive relapsing, *LUTSs* lower urinary tract symptoms, *PDDS* Patient Determined Disease Step

#### Adverse events

There were no reported stimulation-related adverse events in the participants during the study intervention phase. Two (10%) participants reported skin reactions in the site of the stimulation with (*n*=1) redness after first stimulation session and (*n*=1) dry skin at the stimulation site that cleared up after 8 days. This did not impact treatment adherence.

#### Adherence to the treatment protocol

The adherence to the 18 sessions among 20 participants was 100%.

#### Acceptability of TTNS application

The responses to the participant satisfaction questionnaire (Likert scale) are shown in Table 3. The median score was 5 points (IQR=0.25 points) regarding the ease of use of the device, indicating that participants strongly agreed that the device was easy to use. Median scores of 5 points were also recorded for acceptability of the frequency, duration, and timing of the intervention. Overall, participants were very satisfied with the intervention (median score=5 points, IQR=0 points).

**Table 3:** Acceptability (satisfaction) survey (*n*=20)

Questions	Median score (IQR)
Q1. Please, tick the appropriate box that indicates your satisfaction with nerve stimulation - The device is easy to use.	5 (0.25)
Q2. Please, tick the appropriate box that indicates your satisfaction with nerve stimulation - The device is comfortable to use.	4.5 (1)
Q3. Please, tick the appropriate box that indicates your satisfaction with nerve stimulation - application of tibial nerve stimulation at home was acceptable.	5 (1)
Q4. Please, tick the appropriate box that indicates your satisfaction with nerve stimulation - Using the tibial nerve stimulation for 30 mins 3 times/ week is acceptable.	5 (0.25)
Q5. Please, tick the appropriate box that indicates your satisfaction with nerve stimulation - Using the tibial nerve stimulation for 6 weeks is acceptable.	5 (1)
Q6. Please, tick the appropriate box that indicates your satisfaction with nerve stimulation - Overall, I am satisfied with the device.	5 (0)

5-point Likert scale employed across questions (score of 1= “strongly disagree”; 2= “disagree”; 3= “neutral”; 4= “agree”; 5= “strongly agree

### Secondary outcome measures

#### Changes in bladder storage symptoms reported using 3-day bladder diary and PPIUS

Table 4 displays the baseline and post-intervention score across all outcome measures. Daily average urination, urinary frequency in 24 h, changed from a daily median of 10 times (SD=3) to 8 times (SD=3). Median daily urinary urgency changed from 6 times (IQR=5.8) at baseline to 2 times daily (IQR=4) post-intervention. No changes were observed in the daily mean episodes of nocturia (mean=1 episode, SD=1).

**Table 4:** Scores of the outcome measures (*n*=20)

	Baseline	Post-intervention (week 6)
**3-day bladder diary scores**		
Daily average urinary frequency (24 h): mean (SD)	10 (3)	8 (3)
Daily average urgency: median (IQR)	6 (5.8)	2 (4)
Daily average urinary incontinence: median (IQR)	0 (1.3)	0 (0)
Daily average nocturia: mean (SD)	1 (1)	1 (1)
Daily average fluid intake: mean (SD)	1585 (604.5)	1504 (489)
Daily average fluid intake: Caffeinated drinks: mean (SD)	542 (367)	547 (360)
Daily average fluid intake: non-caffeinated drinks: mean (SD)	1043 (566)	957 (491)
**Patient Perception of Intensity of Urgency Scale (PPIUS)**		
Daily average total scores PPIUS: median (IQR)	2 (1)	2 (1)
Daily average severe urgency grade (3): median (IQR)	3 (4)	1 (1)
Daily average urge urinary incontinence grade (4): median (IQR)	0 (0.6)	0 (0)
Daily average severe urgency + urge urinary incontinence grade (3+4): median (IQR)	3 (3.4)	1 (1)
**International Consultation on Incontinence Questionnaire Over active bladder (ICIQ-OAB) scores**		
Total score ICIQ-OAB: median (IQR)	8 (2.25)	4 (2.5)
Q1. Frequency: median (IQR)	1 (2)	1 (2)
Q2. Nocturia: median (IQR)	2 (2)	1 (0.5)
Q3. Urgency: median (IQR)	3 (1)	1 (1)
Q4. Urge urinary incontinence: median (IQR)	2 (1)	1 (1)
**Kings Health Questionnaire (KHQ)**		
Total score: part-one: mean (SD); median (IQR)	5.8 (1.2); 6 (1.25)	5 (1.27)*; 5 (2)*
Total score: part-two: mean (SD); median (IQR)	44.6 (8.9); 43 (10)	28.4 (8.5); 27.5 (8)
Total score: part-three: mean (SD); median (IQR)	10 (2.7)*; 10 (4.25)*	7.55 (4)*; 6.5 (5.25)*

*SD* standard deviation, *IQR* interquartile range

*Indicates that data are not normally distributed

The daily median urgency scores (grade 3) assessed by PPIUS changed from 3 points (IQR=4) to 1 point (IQR=1) post-intervention with no changes were reported in daily median urgency urinary incontinence (UUI). Daily average fluid consumption remained stable across both timepoints.

### Changes in bladder storage symptoms reported using ICIQ-OAB

The ICIQ-OAB median scores are summarised in Table 4. The overall median (IQR) scores for urinary symptoms as measured by the ICIQ-OAB changed from 8 points (IQR=2.25) at baseline to 4 points (IQR=2.5) post-intervention.

### Changes in health-related quality of life scores for urinary symptoms reported by KHQ

The KHQ scores at baseline and post-intervention are summarised in Table 4. No changes in mean/median total scores were reported in the KHQ-Part- one. The median and mean scores of part-two changed from 43 points (IQR=10) and 44.6 points (SD= 8.9) at baseline to 27.5 points (IQR=8) and 28.4 points (SD=8.5) at the end of week 6, respectively. Part-three showed changes from 10 points (IQR=4.25) and 10 points (SD=2.7) at baseline to 6.5 points (IQR=5.25) and 7.55 (SD=4) post-intervention, respectively.

## Discussion

This study demonstrates the feasibility and acceptability of TTNS for bladder storage symptoms in PwMS using an accessible and affordable TENS unit. Our key progression criteria to continue to a pilot randomised controlled trial were achieved. Participants reported high levels of satisfaction with the study protocol and high adherence was reported for completion of the 6 weeks intervention with no serious adverse events reported in the current study.

Adherence to the intervention was high and may have been influenced by weekly follow-up calls. A meta-analysis of 127 included studies showed that the odds of patients’ treatment adherence are 2.16 times higher with effective communication and regular monitoring between healthcare providers and patients [36]. TTNS in the current study showed high level of adherence (100%) compared to a low level of adherence to pelvic floor muscle exercise (PFME) (20%) among women with urinary incontinence [37]. Previous studies showed that healthcare providers estimated that 64% of patients adhere to PFMT in short term, but 23% in long term [38] with limited data in literature on the adherence rate of non-pharmacologic management strategies of urinary symptoms among PwMS. The acceptability of home-based TTNS is supported by a previous study where it was more acceptable than home-based PFME [39]. TTNS is a non-invasive technique that makes home-based treatment accessible compared to PTNS which needs clinic visit for each treatment session [40]. Also, the application of TTNS does not need frequent follow-up from clinicians compared to other interventions including PFME. Studies showed the effectiveness of the PFME depends on regular monitoring provided by frequent assessment of PFM function and contractility which required patients to attend clinic for an individualised training program to achieve optimal outcomes [41]. Furthermore, safety measures of TTNS reported in the current study is in line with previous studies among people with non-neurogenic [42] and neurogenic OAB [14, 43, 44].

We did not seek to examine if participants experienced significant changes in urinary symptoms and quality of life from baseline to post-intervention due to our primary focus on exploring feasibility. However, changes in scores of urinary symptoms using 3-day bladder suggest improvements of urinary frequency and urgency episodes post-intervention of TTNS. In line with our findings, one study [23] found that TTNS at 10Hz, 200ms for 30 min, twice weekly was effective in reducing the urinary frequency and urgency episodes using 3-day bladder diary among post-stroke at the end of 6 weeks of intervention [23]. Specific to PwMS, one study was identified [21] using a 3-day bladder diary which showed that 20 min of daily TTNS for 12 weeks reduced urinary frequency by 2.7 voids after 4 weeks and 2.4 voids after 12 weeks. In the current study, we noticed similar improvements in the reduction of urinary frequency episodes of 2 voids post 6 weeks of intervention. However, the main weakness of our study is the lack of a comparison group due to the nature of the feasibility study.

The current study findings demonstrated a positive trend in reducing the urgency severity symptom scores using PPIUS and reducing urgency episodes, and nocturia using ICIQ with a reduction in scores of QoL measures detected by KHQ indicating improvements of urinary symptoms post TTNS. Similar to current study findings, a reduction in urinary urgency scores following TTNS has been shown among 75 non-neurogenic OAB participants [45], and among a mixed population of 48 neurogenic and non-neurogenic OABs including PwMS [46]. However, the latter study needs to be interpreted with caution because the authors did not provide a separate analysis for MS participants. A minimal clinically important difference (MCID) has been noticed for KHQ as a change of score of ≥3 points in the symptom severity domain and ≥ 5 points in the other domains of the questionnaire [47], suggesting a clinically meaningful change in QoL in our participants. However, direct comparison between our study findings with existing literature is difficult due to the lack of a standardised treatment protocol including a total number of sessions per week, the duration of treatment, and the parameters for stimulation [40, 42, 48, 49] and inconsistency of outcome measures to assess the urinary symptoms in literature. Furthermore, the current study includes participants with any type of MS and the impact of TTNS on urinary symptoms and QoL measures should be interpreted in the context of this heterogeneous group. The use of a randomisation schedule and allocation concealment in future studies should address both known confounding such as MS type, gender etc and unknown confounding.

Remaining uncertainty that needs to be addressed in the randomised pilot trial is as follows:

1. Inclusion of long-term follow-up of the intervention (12 months)
2. Include sham-arm to investigate the effectiveness of TTNS on NLUTD in MS
3. Provision of a face-to-face option rather than online only to widen the recruitment and support MS participants who do not have access to the internet.

### Implications

- TTNS is feasible, acceptable, and safe in clinical practice with PwMS and bladder storage symptoms
- Rates of recruitment and retention to this feasibility study were acceptable
- Future Pilot RCT are warranted to assess the effectiveness of TTNS in PwMS and bladder storage symptoms

## Conclusions

The current study demonstrates that TTNS is feasible, safe, and acceptable to PwMS. Changes of urinary symptoms scores between baseline and post-intervention suggested improvements in bladder storage symptoms and QoL measures. These findings serve as a preliminary step to inform the development of an evidence-based future pilot randomised controlled trial (RCT) using standardised reporting of outcome measures. We recommend following the treatment protocol, the electrical stimulation parameters, and the outcome measures applied in the current feasibility study for the implementation plan of the future pilot RCT.

## Supplementary Information

**Additional file 1.** CONSORT 2010 checklist of information to include when reporting a pilot or feasibility trial*.

## Data Availability

The datasets supporting the conclusions of this article are included within the article (and its additional file)

## Abbreviations

NLUTD: Neurogenic lower urinary tract dysfunction
PwMS: People with MS
DO: Detrusor overactivity
CNS: Central nervous system
EUA: European Association of Urology
NICE: UK National Institute for Care and Clinical Excellence
TNS: Tibial nerve stimulation
TTNS: Transcutaneous tibial nerve stimulation
PTNS: Percutaneous tibial nerve stimulation
TENS: Transcutaneous electrical nerve stimulation
PDDS: Patient Determined Disease Steps
ICIQ-OAB: International Consultation of Incontinence Questionnaire- Overactive Bladder
PPIUS: Patient Perception of Intensity of Urgency Scale
QoL: Quality of life
KHQ: King’s Health Questionnaire
SD: Standard deviations
IQR: Interquartile ranges
RRMS: Relapsing remitting
PFME: Pelvic floor muscle exercise
RCT: Randomised controlled trial
